# The Idealized Brazilian Health System versus the real one: contributions from the nursing field^1^

**DOI:** 10.1590/0104-1169.0040.2512

**Published:** 2014

**Authors:** Dirce Stein Backes, Martha Helena Teixeira de Souza, Mara Teixeira Caino Marchiori, Juliana Silveira Colomé, Marli Terezinha Stein Backes, Wilson Danilo Lunardi

**Affiliations:** 2 Post-doctoral fellow, Centro de Ciências da Saúde, Centro Universitário Franciscano, Santa Maria, RS, Brazil. Full Professor, Centro Universitário Franciscano, Santa Maria, RS, Brazil; 3 PhD, Full Professor, Centro Universitário Franciscano, Santa Maria, RS, Brazil; 4 Doctoral student, Centro de Ciências da Saúde, Centro Universitário Franciscano, Santa Maria, RS, Brazil. Associate Professor, Centro Universitário Franciscano, Santa Maria, RS, Brazil; 5 PhD, Professor, Universidade Federal de Santa Catarina, Florianópolis, SC, Brazil; 6 PhD, Associate Professor, Universidade Federal do Rio Grande, Rio Grande, RS, Brazil

**Keywords:** Nursing Research, Unified Health System, Human Resources Formation

## Abstract

**OBJECTIVE:**

to identify the perceptions of professionals working in a facility connected with the Brazilian Unified Health System - SUS in regard to what they know, think and talk about public health policy.

**METHOD:**

this exploratory-descriptive study with a qualitative nature was conducted with 28 professionals working in a facility connected with the SUS. Data were collected through interviews with guiding questions and analyzed through the thematic content analysis technique.

**RESULTS:**

coded and interpreted data resulted in three thematic axes: The SUS - perfect web that does not work in practice; The recurrent habit of complaining about the SUS; The need to rethink the way of thinking about, acting in and managing the SUS.

**CONCLUSION:**

the professionals working for the SUS are aware of the principles and guidelines that govern the Brazilian health system, however, they reproduce a dichotomous and linear model of conception and practice strongly linked to the thinking of society in general.

## Introduction

The Alma-Ata Declaration of the International Conference of Primary Health Care conducted in 1978 reaffirmed that nations around the world would effectively participate in the health promotion of their citizens through health practices intended to attain physical, mental and social wellbeing, a fundamental right of their inhabitants. From this perspective, health became one of the most important goals of world society and to achieve such a goal, there needs to be cooperation among the diverse social, economic and political sectors^(^ 1 ^-^ 2 ^)^.

Therefore, the Brazilian Constitution of 1988 established that every Brazilian citizen has the right to access primary, secondary and tertiary healthcare that is provided free of cost by a national health system^(^ 1 ^-^ 3 ^)^. Based on this premise, the Unified Health System (SUS) was created with unique characteristics in Latin America. Its implementation resulted in decentralization, and consequently, in increased access for people to healthcare services, especially primary health care, through the Family Health Strategy^(^ 2 ^-^ 6 ^)^.

In this process, Brazil has invested in a universal system centered on primary health care at the same time in which many other countries opted for selective care and other, less equitable, funding strategies. This change has led to an expressive increase of coverage with positive effects in various fields^(^ 3 ^)^. One study conducted in 2008 revealed that 93% of Brazilians seeking healthcare services successfully accessed healthcare and that many interventions in the maternal-infant field are close to achieving universal coverage as ensured by SUS principles^(^ 3 ^-^ 5 ^)^.

Formed by a network of services and health actions provided by federal, state, and city institutions and facilities, the SUS can be considered one of the greatest social achievements consecrated in the Federal Constitution of 1988^(^ 3 ^-^ 4 ^)^. Achieved amid participatory and democratic struggles, the SUS is grounded on principles such as universality, equity, and integrality and is structured based on organizational guidelines such as decentralization, regionalization, hierarchization, and popular participation^(^ 6 ^-^ 9 ^)^.

The SUS is the materialization of an broadened and contextualized expectation of health, due to the possibility of SUS practice transcending the biologicist logic and reaching a conception of health as a social good^(^ 8 ^-^ 9 ^)^. Its creation, was therefore, the greatest movement of social inclusion ever seen in Brazilian history. Since that time, there has been in Brazil intense and expanding advancements in the health sector, but at the same time, great and important challenges are faced both in the management field and in the field of processes and care delivery^(^ 9 ^)^. At the same time, there is a culture that discredits and devalues issues concerning the SUS, both on the part of managers and professionals regarding the system and on the part of the population, who in general enjoy its services. This perception, is in many cases, related to misinformation concerning the principles and guidelines that govern the SUS and also due to the dominance of the biomedical model, still centered in secondary and tertiary care^(^ 2 ^,^ 10 ^-^ 11 ^)^.

It has been asserted that the development of a systemic, proactive form of management that values issues regarding the SUS should begin among its own workers, providing healthcare though theoretical-practical interventions proposing new ways of thinking and acting. For that, continuous education in the health field is an important mechanism to linking learning needs and working needs, considering that continuous education is part of the SUS's purposive roles^(^ 12 ^)^. Continuous education in the health field can be characterized as a methodological proposal of significant learning at work, in which learning and teaching are incorporated into the routines of organizations and into their work itself, to enable the qualification of professional practices^(^ 13 ^)^.

In this process of attaining achievements, nurses have played an increasingly decisive and proactive role, especially in terms of identifying healthcare needs, as well as promoting policies directed to the promotion and protection of the health of individuals, families and communities. Even if interconnected and complemented by other types of professional knowledge, nursing can be largely defined as the science that integrates healthcare, both in the sense of assisting and coordinating care practices and in the sense of promoting strategies directed to continuous education in health^(^ 14 ^-^ 15 ^)^.

This study is linked to a larger project of continuous education in the health field. *A priori*, however, we opted to investigate what professionals think and talk about in terms of the SUS, so at a second point in time, we also opted to intervene in this context with a proposal of continuous education. Hence, this study's aim was to identify what professionals working in a facility connected with the Unified Health System know, think and talk about the public policy of health. 

## Method

This exploratory-descriptive study used a qualitative approach because of its importance in the field of social and health investigation, particularly in the context of studies focusing on symbolic dimensions and demanding deeper understanding, analysis and impact assessment^(^ 16 ^)^.

Data were collected through interviews between November and December 2013 on days and at times previoulsy scheduled with the workers of a facility connected with the SUS, located in the central region of the state of Rio Grande do Sul, Brazil. The interviews were guided by the following questions: Please tell me what you think about the SUS. As a professional working in this instituiton, how do you perceive the work dynamics in the context of the SUS?

The informants were randomly chosen and formaly invited in the workplace. Of the 30 professionals invited, 28 consented to participate in the study and signed free and informed consent forms. Nurses, dietitians, pharmacists, nursing technicians and support professionals from the management, hospitality, office, pharmacy and nutrition sectors composed the sample.

The interviews were transcribed and the empirical material was submited to content thematic analysis^(^ 17 ^)^ in order to identify core meanings that compose communication, the presence or frequency of which added significant perspectives to the object of study. The notion of this thematic treatment was associated with recurrent statements expressed by words, phrases or ideas. Hence, the operationalization of this process followed the three stages recommended to conduct a thematic content analysis.

Exhaustive reading of data was performed in the first stage, pre-analysis, followed by the organization of material and the formulation of hypotheses. Afterwards, the material was explored, that is, raw data were coded. In the third and final stage, data were interpreted and thematic axes were concomitantly delimited based on the understanding of the meanings established^(^ 17 ^)^.

To comply with ethical criteria, we folowed the recommendations of Resolution No. 466/2012 from the Brazilian Council of Health, which regulates studies involving human subjects^(^ 18 ^)^. The project was submited to and aproved by the Institutional Review Board (No. 308.493/2013). To maintain the confidentiality of the participants, the reports are idnetifiied in the text by the letter "P" followed by the number corresponding to the order in which the interviewes were conducted. 

## Results

Coding and interpretation of data resulted in three thematic axes, namely: The SUS - perfect web that does not work in practice; The recurrent habit of complaining of the SUS; The need to rethink how you think about, act in and manage the SUS.

### The SUS - perfect web that does not work in practice 

All the workers' reports make clear that the SUS is a perfect web/network, the best national health system and an example for other countries. They recognize that it reaches different economic classes and comprises from the basic to the most complex services, provided free of cost. Therefore, it is a system that had been improved through new programs and policies but which, in practice, presents operational obstacles.

In this antagonism and dialectical perception, the informants mentioned that difficult access and delay experienced in the services are the main obstacles hindering the proper functioning of the SUS in practice: *The* *SUS is a perfect web. If it worked in practice, it would be perfect. The United States came here to take it by example. It's difficult for you to enter into the system, but once you get in, you can do everything. You can have surgery if you need to (P1).*

At the same time in which the interviewees mentioned difficulties related to delay and difficult access to the service, they recognized that they cannot imagine Brazil without the SUS. *I can't imagine Brazil without the SUS. Even though it's a poor and deficient system, I can't imagine it (P13). *Again, the workers show an antagonist relationship. At the same time the system is *poor and deficient*, it's also part of the history and lives of Brazilians, i.e., it has been internalized as a social good. On the other hand, some workers cannot find an explanation for the delay in the performance of exams within the SUS or the need to wait in a line throughout the night in order to ensure an opportunity to get a consultation.* Sometimes we perform surgeries to remove a small mole while there are people who scheduled a consultation one year before. I don't know why there is so much delay (P5). My husband went to a health unit two years ago to schedule a consultation with a neurologist. A few days ago, they called him to get an exam but, during this time, he already had his ischemia. I'd like to understand why that happened that way. Not to mention the need for people to go at 4 in the morning to get a number for a consultation. I don't understand it (P26).*

In addition to the problems concerning access and delay mentioned by all the interviewees, some criticized political interventions, the deviation of public funds, and a lack of systemic cooperation among the diverse sectors. They reaffirm that the SUS is a perfect web but, in practice, the wires/sectors do not connect or even intersect, i.e., there is no communication among them to find joint solutions. *I don't agree with the idea of having trusted political appointees in the health sector. Positions should be occupied by those with the knowledge, with the background to manage this network (P1). Sometimes I feel there is no desire on the part of professionals. They are not united. The health unit does not cooperate with the hospital. When we try, we see people have an adverse reaction. I try to understand why this resistance exists, but I can't. It seems it's a game and no one wants to assume the responsibility, you know? (P8). The network doesn't work and I say so having experienced it myself. Sometimes, the person is discharged from hospital but doesn't receive follow-up; the primary healthcare network works very poorly... (P9).*

In general, the participants are aware of the principles and guidelines governing the SUS and acknowledge its importance for the health of Brazilians. They also show a reflective process upon the questions presented and try to understand why the delays exist, why patients need to remain in lines throughout night to ensure they will get a consultation, that is, why there are these barriers that would apparently be possible to solve. 

### The recurrent habit of complaining about the SUS 

At the same time the workers mentioned that difficult access and delay in the delivery of health services are obstacles resulting from management of the service and organization, they perceive that such assumptions result from a cultural process, translated into the habit of complaining: *There's this habit of complaining, complaining, complaining. We complain about everything... sometimes it's even... (P27).* The participants' reports show that some complain because they had to wait in line; others complain because there were not enough physicians in the PHC unit. Still others complain because they did not receive proper service, while others complain because they lack knowledge concerning the SUS or because the SUS is, by itself, reason enough to complain. *You can do everything within the SUS... There're people who complain but they are very ungrateful. There's this bottleneck in the network, but people are used to complain about everything (P11). Most people I know complain, but they do not understand how the SUS works. Many people do not (P2). Few people agree that the SUS works because they don't have knowledge about it (P10).*

Some reports make it clear that the culture of complaining has always been part of the Brazilian culture. They realize that the SUS has always been subject to criticism, complaints and defamation, with or without reason, and people seldom recognize its benefits. One worker specifically mentioned that *There's this habit of complaining. Ah, too much complaining but it's been like this since forever (P6). *Other interviewees also mentioned that when people complain, they indirectly refer to workers or managers who, for some reason, did not provide proper care or did not make proper referrals. They also highlighted that there is a culture of complaining about everything and everyone, even among the workers themselves. *The patient complains and complains, often with a reason, but because of the attitude of some worker. We also badmouth each other and complain all the time. Why do we have to be like this? (P17).*

Worker themselves are aware that there is a habit of complaining and that this attitude generates and snowballs, in which everyone complains without knowing what they want to achieve by complaining. On the other hand, the culture of complaining about everything and everyone may be a way of the collective to express that there is a "critical knot" in the system, that there is something out of sync. *Why does everyone complain since forever? (P6).*

### The need to rethink they way we think about, act in and manage the SUS 

Most reports show a need to promote a new way of thinking and a new management of the SUS. The interviewees acknowledge that it is necessary to transcend difficulties of access and delay, as well as the habit of complaining about everything and everyone and to propose a new way of thinking in healthcare. *I believe we have to change the way we think about the SUS. Those working for the SUS need to change. There is this mentality that the SUS has to be bureaucratic. I don't agree with this or why some deliver care to only four patients a day; it can't be! That I can only provide service to 20 patients. If I'm hired to work 30 hours a day I have to provide care to the maximum number of patients I can within those 30 hours (P1).* This report clearly shows that not all the professionals are effectively involved in or committed to the system.

A new contradiction that appears in the reports is related to health plans complementary to insurance and the SUS. At the same time that professionals acknowledge that the SUS is the best system and a model for other countries, personally they opt for a private health plan. *I use the SUS but I have a private health plan. I use the SUS to get medication that is too expensive... because the physician here sees you for ten minutes but in my private plan the physician sees me for half a hour (P22).* The workers defend the SUS by asserting that people need to change the way they think about the SUS and the way they manage it, but then they seek the assurance of healthcare delivery in the supplementary health system.

This contradictory, and at the same time, incomprehensible relationship, is partly associated with a conception that the SUS is for the poor and the needy. *There are people here who say: 'Ah! Have you seen that inpatient? Chic and well-dressed, you can tell she has money. Why is she hospitalized using the SUS?' It seems that the SUS is visible. People say anything. It's a lack of knowledge (P17). The SUS is to take care of the needy, you know? But there is a lot of middle and upper class people who use the SUS, including to get free-of-cost immunization (P23).*

Another element noted by the interviewees is SUS management and/or its managers. They acknowledge that many managers are trusted political appointees and occupy and management positions without proper qualification or competence to perform the function. *A factor that needs to change is the qualification of managers. As I told you before, if the mayor appoints someone to be the secretary of health, this person has to have at least... I think it's very trivialized... there're trusted political appointees ...I think they should have minimum knowledge in the field... It'd be a great thing (P11). I think that right now, the SUS is missing management, because if the law was implemented, it would work for real; it'd be different. Management is what's missing (P13).*

Changing the way of thinking about and managing the SUS, according to the professionals' reports, implies rethinking the system's management process, the conception that the SUS is a universal system and not only for the poor and the needy. The understanding of the system as effectively a web/network that involves dialogue, interaction, and the commitment of all those involved in the process is essential. These reports also show that both workers and users need to be engaged in and committed to the system. For that, we need to transcend specific issues of four consultations a day or providing 20 forms daily and conceive of the system as an integrated whole, so this web/network will actually work in practice. 

## Discussion

The SUS is by itself a complex phenomenon. Thus, the more complex will the discussion be concerning a search to outline new theoretical abstractions for the system to truly work in practice. Therefore, only a framework that is also complex will enable a new understanding and new strategies and avoid theoretical-practical simplifications. 

Some questions that could immediately initiate the discussion process emerged: Why are there access and delay barriers in the system? Where is the beginning or the cause of such a phenomenon? Who are the main people responsible for putting this complex and systemic gear into operation? What is needed and can actually be accomplished for this systemic and circular gear to be synchronized for its effective functioning? What is the role of nursing in this systemic configuration?

It is worth noting that, *a priori*, the SUS is composed of a health system with a strong connotation of disease. When the system is conceived as disease-centered and/or focused on the needs presented by a specific disease, one needs to ask: how long would I wait in life to have access and/or to receive treatment in a situation of pain and/or suffering? What would I consider to be an acceptable length of waiting for someone in my family to receive care if in this same situation? Hence, when we reflect upon these and other issues, it is understandable that many workers seek and pay for a health plan to ensure they receive health care and/or to avoid the situations previously mentioned.

This same idea however, opens up space for new questions: If the SUS is moved and operationalized by people, why do the workers themselves not believe in, qualify or have confidence in their work and instead seek a supplementary health plan? What is the difference between professional work within the SUS and the work provided within the private sector? Finally, why do they not believe or not want to believe in the SUS? Why is the SUS considered to be for the poor and needy, if according to the law, access is ensured to all citizens? Why does this habit of complaining about and speaking ill of the SUS exist?

Another aspect noted by many professionals refers to the waiting-number patients need to get during the night to ensure they get a consultation. We ask: Would I leave my child with a fever at home to ensure I get a number in line during the night? Why do many opt for and/or seek an emergency care unit, which ideally, would not serve as the entrance door to the system, to ensure they receive healthcare? This idea became clear in the reports of many workers, as they emphasize that many people prefer seeking care in an emergency department instead of waiting in lines in the middle of the night when there is a risk of not having a physician to see them at the scheduled time. Why does the system need to work this way? Is this a problem of the system and/or of the linear and simplified perception of those who operationalize it?

Reflecting upon these and other questions that may come to mind is the first step to proposing a new way of thinking about and managing the SUS without, however, the pretension of giving pat answers. Today, more than ever, it is urgent to rethink being and doing within the SUS. For that, a complex and systemic way of thinking may be capable of discussing and problematizing such issues. 

The SUS is by itself a complex unit, but the thinking of people, in general, remains simplified, fragmentized and reductionist. Thus, we are talking of new theoretical frameworks, new ways of thinking, a new culture able to understand the SUS in an enlarged, integrated and connected manner, with different types of knowledge, services and sectors that compose it. The need to think in a new framework is corroborated by many authors of studies previously conducted in the field^(^ 11 ^,^ 13 ^,^ 19 ^-^ 20 ^)^. But, how can we promote this new way of thinking about and managing the SUS?

Traditional and hegemonic management models of conceiving the SUS have been increasingly questioned and challenged in the current context, in light of the complex thinking about the topic. It is a framework that enables the construction of dynamic and circular networks, the promotion of multidimensional knowledge, as well as critical, reflective processes committed to the whole - the SUS system. From this perspective, complex thinking supports the idea that we need to overcome a naïve consciousness and achieve a critical consciousness, to acquire a perception of the living world as a network of multiple relationships. Therefore, it is important to overcome the habit of complaining and achieve a conscious, proactive and innovative attitude. Hence, managers need to be apt to integrate, rewire and energize the SUS - a complex web/network, in which different wires need to meet and form a complex unit^(^ 21 ^-^ 22 ^)^.

By complex, we mean everything that is decided together^(^ 21 ^)^. In this direction, the complexity originates from a tangle of interactions, associations, feedback, events, incidents, which altogether constitute different social phenomena, in this case, the SUS. Complex thinking does not presuppose the elimination of simplification and does not have the pretension of being complete, but is presented as a path, capable of enabling a new understanding and new ways of thinking about and managing the SUS^(^ 22 ^-^ 24 ^)^.

Complex thinking enables, by its nature, a reflective attitude on the part of a subject about him/herself and how to act in society, in this case, in the SUS. In this sense, reflectivity enables the transposition of linear, reductionist thinking, imposed by established knowledge, into the adoption of new ways of thinking and acting in the system as a whole. The new thinking about and managing the SUS in light of complex thinking involves care practices that are conceived as an intricate relationship of wires that intersect in a web, that is, in a plural and multidimensional web of professional types of knowledge, services, sectors and others. Each area of knowledge remains important in this web but its relevance is in its ability to interlink and interconnect it with the other types of knowledge in order to make the SUS as a complex and dynamic unit^(^ 11 ^,^ 15 ^)^.

In the process of gaining achievements and facing challenges related to the SUS, nursing has the opportunity to operate proactively and systemically at the different levels of healthcare, whether through education, promotion or rehabilitation of health. This operation particularly occurs in an effort to raise critical situations and the systematized intervention of a healthcare plan, capable of overcoming fragmentation and ensuring the continuity of healthcare and its problem-solving capacity^(^ 20 ^)^.

## Conclusion

The conclusion is that SUS workers have a superficial knowledge of the principles and guidelines that govern the Brazilian healthcare system. The participants reproduce a model of healthcare conception and dichotomous, occasional and linear performances in their practices. For the principles and guidelines to be translated into practice, it is inevitable that new theoretical-practical frameworks be proposed. 

The study shows that a health system that can serve as a model for international countries or is a perfect web, as expressed by the workers in it, is not enough. It is necessary that this system be translated into practice, i.e., into thinking about, acting in and managing healthcare. For that, transcending the simplifying and linear thinking of understanding the SUS is necessary and then conceiving a complex way of thinking, capable of integrating and interconnecting the different types of knowledge, services and health sectors.

From this perspective, continuous education in health is presented as a dynamic device that energizes more affirmative professional attitudes in regard to the development of the SUS. The involvement of users through appropriating the proposal that guides this system, as well as their rights and duties, is also shown as an element with potential to enable new forms for analysis and to make the system more dynamic.

Nursing assumes, in this process, an increasingly decisive and proactive function of identifying the population's care needs as well as promoting and protecting the health of individuals in their multiple dimensions. In short, nursing care is a key component in the local health system, which are reflected at the regional and national levels, and therefore, also presents a motive for encouraging debates and new meanings. 
